# Mechanochemically-Activated Solid-State Synthesis of Borate-Substituted Tricalcium Phosphate: Evaluation of Biocompatibility and Antimicrobial Performance

**DOI:** 10.3390/molecules30071575

**Published:** 2025-03-31

**Authors:** Daniil O. Golubchikov, Inna V. Fadeeva, Alexander V. Knot’ko, Iliya A. Kostykov, Tatiana K. Slonskaya, Katia Barbaro, Alessia Zepparoni, Marco Fosca, Iulian V. Antoniac, Julietta V. Rau

**Affiliations:** 1 Chemistry Department, Lomonosov Moscow State University, Leninskie Gory 1, 119991 Moscow, Russia; alknt@mail.ru (A.V.K.); elikosst98@gmail.com (I.A.K.); 2 Department of Materials Science, Lomonosov Moscow State University, Leninskie Gory 1, 119991 Moscow, Russia; 3 A.A. Baikov Institute of Metallurgy and Material Science, Russian Academy of Sciences, Leninsky 49, 119334 Moscow, Russia; fadeeva_inna@mail.ru; 4 Department of Analytical, Physical and Colloid Chemistry, Institute of Pharmacy, I.M. Sechenov First Moscow State Medical University, Trubetskaya 8, build. 2, 119048 Moscow, Russia; slonskaya_t_k@staff.sechenov.ru (T.K.S.); giulietta.rau@ism.cnr.it (J.V.R.); 5 Istituto Zooprofilattico Sperimentale Lazio e Toscana “M. Aleandri”, Via Appia Nuova 14111, 00178 Rome, Italy; katia.barbaro@izslt.it (K.B.); alessia.zepparoni@izslt.it (A.Z.); 6 Istituto di Struttura della Materia, Consiglio Nazionale delle Ricerche, ISM-CNR, Via del Fosso del Cavaliere 100, 00133 Rome, Italy; 7 Faculty of Material Science and Engineering, National University of Science and Technology Politehnica Bucharest, 313 Splaiul Independentei, District 6, RO-060042 Bucharest, Romania; iulian.antoniac@upb.ro; 8 Academy of Romanian Scientists, 54 Splaiul Independentei, RO-050094 Bucharest, Romania

**Keywords:** bone tissue engineering, tricalcium phosphate ceramics, borate-substituted tricalcium phosphate, antimicrobial properties, biocompatibility

## Abstract

Current research in bone tissue engineering is focused not only on basic parameters of the materials, such as biocompatibility and degradation rate but also on intrinsic osteogenic and antimicrobial properties, essential to provide a rapid tissue regeneration without negative effects due to periprosthetic infections, that may result in revision surgeries. One of the major strategies to enhance the osteogenic and antimicrobial performance of calcium phosphates is the ionic substitution, in particular, with magnesium and borates. In this study, we focused on the synthesis of boron-substituted tricalcium phosphate (B-TCP) with a target of 5 mol.% substitution via the solid-state synthesis with mechano-activation. Synthesis from raw precursors, without the preliminary brushite wet precipitation, led to the primary phase of β-TCP, which was proved by the XRD analysis. According to the IR-spectroscopy and ^31^P NMR analysis, boron substitution occurred in the synthesized sample. The developed material showed a modest antibacterial performance against *E. coli*, with 13.5 ± 5.0% growth inhibition, and *E. faecalis*, with 16.7 ± 5.5% inhibition. The biocompatibility of β-TCP and B-TCP was tested through the MTT assay and osteogenic differentiation of the mesenchymal stromal cells. The proposed synthesis approach can be useful for the fabrication of B-TCP ceramics for bone tissue engineering.

## 1. Introduction

State-of-the-art materials for bone tissue engineering are designed to meet bioresorbability requirements within the human body, a fundamental principle of regenerative medicine [1,2]. Calcium phosphates (CPs) keep the leading position in both clinical applications and cutting-edge research studies due to their relevant in vivo osteogenic activity dependent on the Ca/P ratio [3]. CP-based bioactive ceramics can be utilized in a form of bulk and porous scaffolds, or coatings on metals or polymer-based bone implants [4,5,6].

Stoichiometric hydroxyapatite (HAp), which is structured closely to the biological apatite, has received extended interest for bone tissue scaffold fabrication [7]. However, it lacks resorbability, which is strongly correlated with the Ca/P ratio [8]. With the reduction of the Ca/P ratio down to 1.5, which corresponds to tricalcium phosphate (TCP), the biodegradation rate/solubility increases significantly. Thus, tricalcium phosphate also became a conventional material for bone tissue engineering applications since it provided an enhanced biodegradation rate and bioactivity compared to HAp [9].

Emerging materials for bone restoration are required to exhibit an osteoinductive potential, inducing the ability to form native bone. Among the approaches that supported the materials’ osteoinductive behavior, the introduction of additional ions into the calcium phosphate structure [10,11,12] deserves attention. Magnesium, silver, and lithium were shown to induce osteogenic differentiation with further osteoblast proliferation, while silver, lithium, and aluminum promoted mesenchymal stem cell proliferation [11,13,14]. Furthermore, zinc and strontium, alongside calcium, acted as stimuli for the new bone formation [11,15]. Another important process occurring during bone regeneration is vascularization, which can be promoted via the introduction of manganese, cobalt, or copper [11,16].

Another challenge in bone tissue engineering arises from periprosthetic infections, which significantly complicate the bone healing process, often leading to revision surgeries and prolonged tissue regeneration. In particular, several bacterial species have been identified as major contributors to periprosthetic joint infection (PJI). *Staphylococcus aureus* (*S. aureus*), a Gram-positive bacterium, is among the most prevalent pathogens responsible for PJI. In contrast, Gram-negative bacteria such as *Pseudomonas aeruginosa* (*P. aeruginosa*) are associated with a lower number of cases [17,18].

Some of the previously mentioned cationic dopants, including silver [19], strontium [20], and manganese, exhibit antibacterial properties; thereby, the doped calcium phosphate ceramic implementation can prevent bacterial activity and biofilm formation. However, some of them showed considerable toxicity in the in vitro tests [21,22,23]. Moreover, the toxicity of these elements in the long-term in vivo studies is still unrevealed. Another element found in perspective for the production of substituted CPs with antibacterial properties is boron, which was involved in studies regarding antimicrobial therapy and revealed to show high antibiofilm activity [24,25]. The mechanism of boron-contained materials-induced bacterial growth inhibition is still under study. However, it can be partially attributed to the pH changes during the biodegradation process [26,27]. It is worth noting that recent studies revealed a sufficient antibacterial potential of boron-contained bioceramics/bioglass against both Gram-negative and Gram-positive bacteria [28,29]. As previously mentioned, the main limitation of cationic substituted materials was considerable toxicity. While the toxicity of boron-containing bioceramics was also found to be dosage- and time-dependent, cell viability on the 1% boron-containing bioceramic samples was higher compared to Zn, Cu, and Sr-containing materials [27,30,31]. This trend can be explained by the different solubility of doped calcium phosphates, which led to different release profiles of boron and other elements, but it still requires experimental verification.

There are several studies that developed paths to synthesize boron-substituted hydroxyapatite involving both sol-gel and wet precipitation [32,33]. Our previous study aimed at synthesizing monophase boron-substituted tricalcium phosphate (B-TCP) via the precipitation from the aqueous solution; however, it resulted in two-phase samples containing tricalcium phosphate and calcium pyrophosphate [34]. In the present study, we used a mechanoactivation-assisted method of synthesis, which is applied to obtain various substituted calcium phosphates, including both anion- (SiO_4_^4−^) and cation-substitutions (Ca^2+^, Zn^2+^, Mn^2+^, Co^2+^, and Ni^2+^) [35]. Silicate substituted HAp was obtained with the silicate content up to P/Si = 11 [36,37].

Therefore, the present study aimed at synthesizing the boron-substituted tricalcium phosphate via the solid-state synthesis approach from the mechano-activated powders and assessing the biocompatibility and antibacterial properties of the obtained materials. We developed a novel solid-state approach to the B-TCP synthesis with prior mechanoactivation and obtained monophase B-TCP powders. The biological assessment revealed a positive effect on mesenchymal stromal cell proliferation and osteogenic differentiation in the presence of B-TCP. Moreover, modest antibacterial performance has been shown on *E. coli* and *E. faecalis* bacterial strains.

## 2. Results and Discussion

Earlier studies regarding the fabrication of boron-substituted calcium phosphate ceramics have been focused on HAp ceramics [38,39,40]. Recent papers reported the synthesis of more resorbable boron-substituted tricalcium phosphate. However, the prepared samples were heterogeneous, containing TCP and calcium pyrophosphate phases [34]. Moreover, it was difficult to determine the exact phase in which boron was introduced. Therefore, we have developed a new approach for the formation of monophase B-TCP. Two precursor sets were used to synthesize boron-substituted tricalcium phosphate (B-TCP). BTCP-1 was produced via solid-state synthesis at 1150 °C for 3 h, following mechanoactivation with corundum balls. BTCP-2 synthesis involved two stages: brushite was precipitated from calcium hydroxide and ammonium phosphate solutions and then dried. The resulting powder was then reacted with calcium carbonate at 1150 °C for 3 h, followed by mechanoactivation under the same conditions.

**Figure 1:**
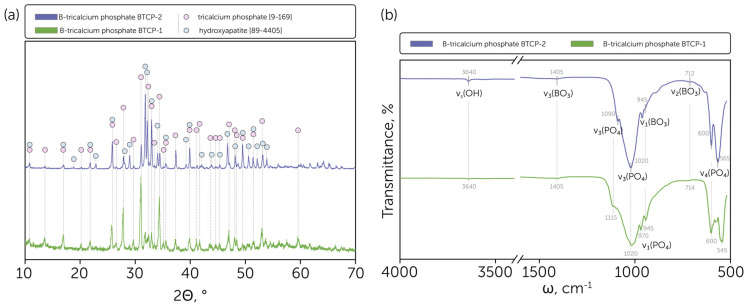
(**a**) Phase composition of the obtained B-tricalcium phosphate BTCP-1 and BTCP-2 powders with indicated tricalcium phosphate [9-169] and hydroxyapatite [99-4405] phases peaks; (**b**) IR spectra of the obtained B-tricalcium phosphate BTCP-1 and BTCP-2 powders with indicated characteristic line positions.

Both synthesized powders contained β-TCP, according to the obtained XRD results (Figure 1a). However, the HAp phase was also detected in both samples with a different content percentage, calculated by the corundum numbers method. As presented in Figure 1a, the B-TCP powder obtained from raw precursors (calcium oxide and ammonia phosphate dibasic) and calcium carbonate without a preliminary wet brushite synthesis had only a minor HAp impurity that did not exceed 5 wt.% of the overall content. In comparison, recent studies reported 30 wt.% [34]. In contrast, the use of brushite and calcium carbonate as precursors led to a significant presence of the HAp phase, which was estimated at about 70 wt.%, resulting in biphasic HAp-TCP ceramics, similar to previous studies [41]. The obtained divergence between the resulting phases arose from the different melting points of precursor phases. In the case of B-TCP-1, during the sintering of the precursor mixture containing ammonia phosphate dibasic, the melting process occurred in the temperature range of 150–225 °C [42] with further melting of boron oxide at 450 °C [43]. Both processes made a significant contribution to homogenization, which led to the Ca/P ratio establishment at a level close to 1.5 and the tricalcium phosphate formation. In contrast, during the solid-state synthesis of the B-TCP-2 sample, the gradual phase transformation occurred without melting: brushite decomposed to monetite and further to the γ-calcium pyrophosphate, which subsequently transformed to the β-calcium pyrophosphate [12,44].

Moreover, the β-TCP diffraction peaks shifted negligibly compared to the ICDD card [9-169]. The effect was supposed to be related to the BO_3_^3−^ ions’ introduction to the β-TCP lattice, replacing the PO_4_^3−^ ions.

The FTIR spectra proved the formation of the primary HAp phase in the powder obtained from brushite and calcium carbonate by the relatively intensive line occurrence at 3640 cm^−1^, which is the characteristic line of HAp related to the presence of hydroxyl (O-H bond stretching) groups in the lattice (Figure 1b). In contrast, the relative intensity of such a bond at the FTIR spectrum of BTCP-1 was insignificant, which correlates with the XRD data. In addition, the BTCP-2 spectrum had a relatively strong 1090 cm^−1^ line, which is the specific ν_3_ PO_4_ line position for the HAp phase; otherwise, for β-TCP, the shoulder line appeared at this position, as presented for the BTCP-1 sample. The 970 cm^−1^ bond also appeared specifically for the BTCP-1 sample and corresponds to the ν_1_ PO_4_ line. Moreover, the ν_4_ PO_4_ lines of β-TCP represented at the BTCP-1 spectrum were shifted to the lower wavenumbers compared to the HAp-related lines [45].

For both BTCP-1 and BTCP-2 samples low-intense ν_3_ BO_3_ lines were found at 1405 cm^−1^ and ν_2_ BO_3_ lines at 714–712 cm^−1^, what supports the estimation of the BO_3_^3−^ introduction to the Ca_3_(PO_4_)_2_ lattice [46].

**Figure 2:**
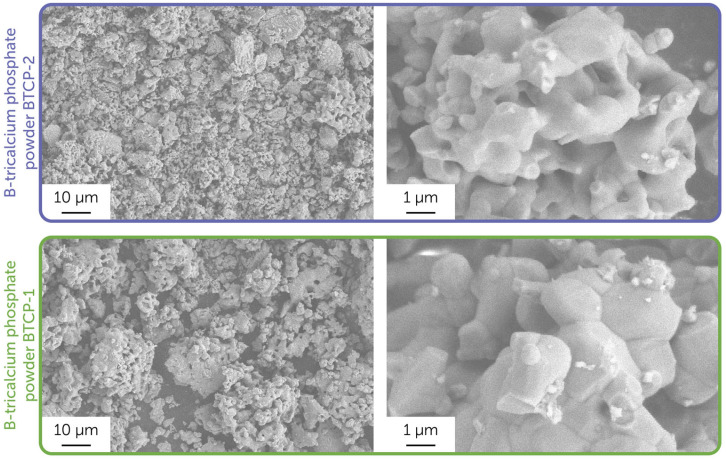
Microstructure of the obtained powder samples, BTCP-1 and BTCP-2. Images were obtained using a secondary electron detector.

Figure 2 presents the microstructure of the obtained powders. Solid-state synthesis at 1150 °C resulted in 2–10 µm particles composed of several sub-micron agglomerates of initial particles. The grain microstructure of the BTCP-1 powder was more visible, and the grains were larger compared to the BTCP-2 powder microstructure.

**Figure 3:**
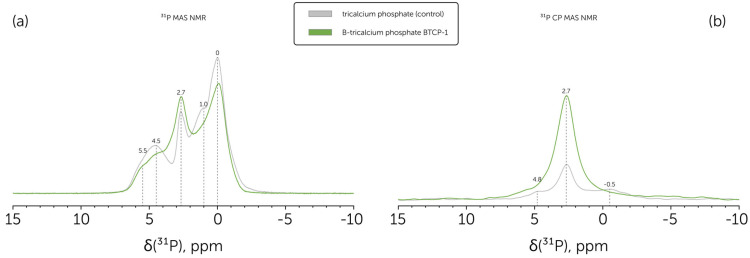
(**a**) ^31^P MAS NMR spectra of the B-tricalcium phosphate BTCP-1 powder (green line) in comparison to control pure tricalcium phosphate powder (grey line); (**b**) ^31^P CP MAS NMR spectra of the B-tricalcium phosphate BTCP-1 powder (green line) in comparison to control pure tricalcium phosphate powder (grey line).

Following the phase analysis provided on the basis of XRD and FTIR, we conducted the ^31^P MAS NMR (Figure 3a) and ^31^P CP MAS NMR (Figure 3b) analysis to reveal the composition of the obtained BTCP-1 powder, corresponded to the β-TCP primary phase, and prove the BO_3_^3−^ ions incorporation to the β-TCP, since this would be hardly determined for the BTCP-2 powder, that had the significant HAp content. The same was accomplished specifically for boron-substituted HAp powders in the previous study conducted by Ternane et al. [46].

Based on Grigg et al. [47], obtained ^31^P NMR peaks can be assigned to pure β-TCP. Namely, the 0 ppm peak corresponded to P(3)Ca(5) coupled sites, which had the highest relative intensity. Each of P(1) and P(2) had two magnetic environments due to the partial Ca(4) site occupancy, which led to four peaks arising. 5.5 and 4.5 ppm peaks were assigned to P(1)Ca(4) and P(2)Ca(4) sites, while 2.7 and 1.0 pp to P(1)V(4) and P(2)V(4), correspondingly. A slight 2.7 ppm peak asymmetry can be explained by a small HAp presence in the studied powders, which had the main ^31^P NMR peak at this chemical shift value [48].

Boron substitution in calcium phosphates was reported to increase peak widths at ^31^P NMR spectra, as observed for the B-TCP sample prepared at 1150 °C. The same trend was presented at the ^31^P CP MAS NMR spectrum. In addition, there was a significant drop in the relative intensity of the P(2)V(4) peak.

**Figure 4:**
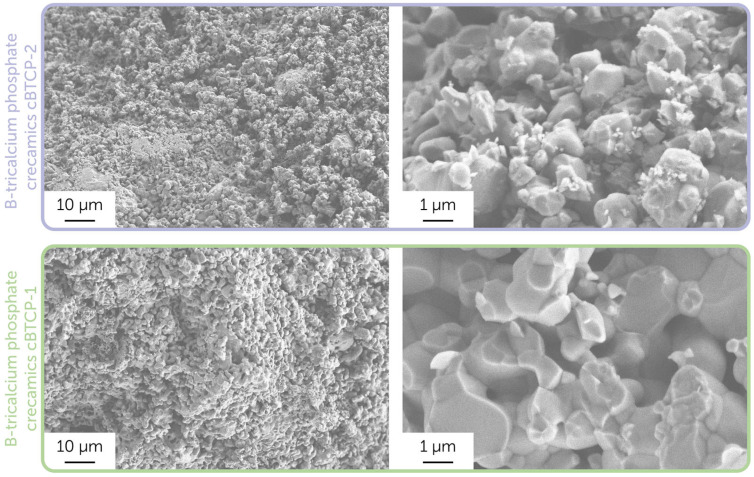
Microstructure of the cBTCP-1 and cBTCP-2 ceramic samples. Images were obtained using a secondary electron detector.

Further, we used synthesized powders to obtain dense ceramic pellets after sintering at 1100 °C. The microstructure of the chipped side of obtained ceramic samples is presented in Figure 4.

As displayed in SEM images related to the biphasic cBTCP-2 sample, there were two types of grains clearly determined: smaller HAp grains located on the larger TCP grains’ edges [49]. The densification rate in monophase TCP ceramics was generally higher, while in composite HAp-TCP samples the slower grain growth was observed due to constrained sintering, led to the large TCP grain growth inhibition on smaller HAp grains. Both final ceramic samples had a sufficient microporosity, resulted from the intensive release of gases during sintering.

It is worth noting that the density of obtained ceramics can significantly affect the cytotoxicity and antimicrobial performance, as denser samples tend to have a lower biodegradation rate, resulting in a lower bioactive element release (e.g., boron). In particular, the ceramics prepared in the current study are dense compared to the previously obtained boron-contained tricalcium phosphate-calcium pyrophosphate ceramics and biphasic hydroxyapatite-tricalcium phosphate ceramics, obtained at the same conditions [50]. Apparently, the introduction of boron has improved the densification process due to the presence of fusible (T_m_ = 450 °C) boron oxide, despite the earlier studies mentioning the opposite trend [24].

**Figure 5:**
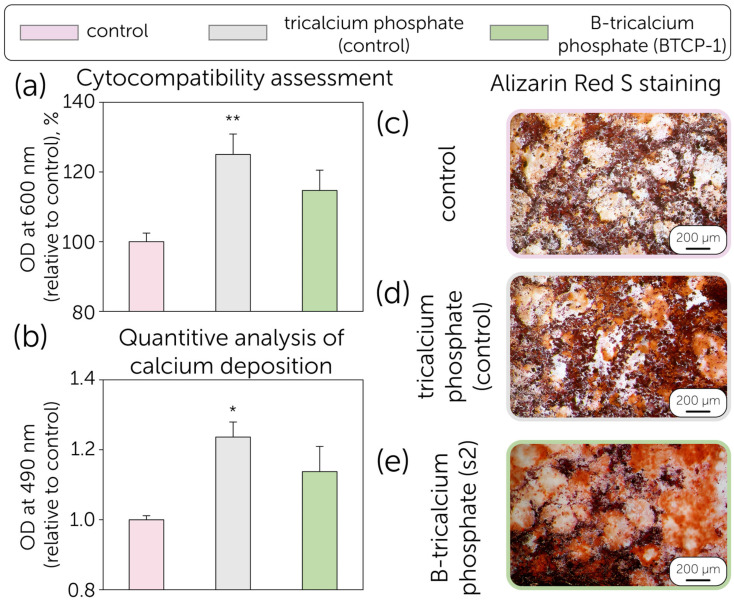
(**a**) MTT Assay: Growth percentage of MSCs cultured for 24 h in the presence of TCP and B-TCP (BTCP-1) compared to the control (cellular control). The values are derived from three experiments and expressed as mean percentage values ± S.D. The values for the cellular control represent 100% growth; (**b**) Quantification of Alizarin Red S on MSCs differentiated into the osteogenic lineage in the absence (positive control) and presence of TCP and B-TCP substrates. The values reported are from three independent experiments and expressed as mean percentage values ± S.D. The values for the positive control correspond to 1. *p*-values (Dunnett test): *, *p* < 0.05; **, *p* < 0.01, relative to the positive cell control; MSCs differentiated into the osteogenic lineage after Alizarin Red S staining. (**c**) Positive control (MSCs with differentiation medium only). (**d**) MSCs differentiated with differentiation medium and TCP, (**e**) MSCs differentiated with differentiation medium and B-TCP. The images were obtained using an inverted optical microscope at 10× magnification.

The MTT assay was used to evaluate the toxicity of TCP and B-TCP, synthesized from raw precursors (BTCP-1) and containing tricalcium phosphate as a primary phase. Figure 5a illustrates the growth percentage and standard deviation (SD) obtained from the mean OD600nm of three experiments.

Despite several recent studies revealing the toxic effect of boron, our data indicate that the growth of MSCs in the presence of TCP is 125.0%, while the growth in the presence of B-TCP is 114.7% compared to the positive control (100%). In both experimental conditions, no toxicity was observed; on the contrary, the growth of MSCs is enhanced in the presence of TCP with statistical significance (*p* < 0.01 **). The cytotoxic effect of boron has been discussed as dependent on the concentration and release profile, which correlates with the solubility and active surface area (porosity) of the ceramic material. However, even 0.5–2% of boron concentration in biphasic hydroxyapatite-tricalcium phosphate ceramics was revealed to negatively affect the material’s cytocompatibility. In comparison, the current research revealed the positive effect of 1% boron concentration on the MSCs proliferation, similar to boron-contained glasses [41,51]. The discussed controversy is related to the differences in density, as more dense ceramics impede the rapid boron release at the early stages of material resorption. The same trend was shown for tricalcium phosphate-calcium pyrophosphate ceramics, which had lower MSC viability due to the lower density and the presence of a more soluble calcium pyrophosphate component [34].

**Figure 6:**
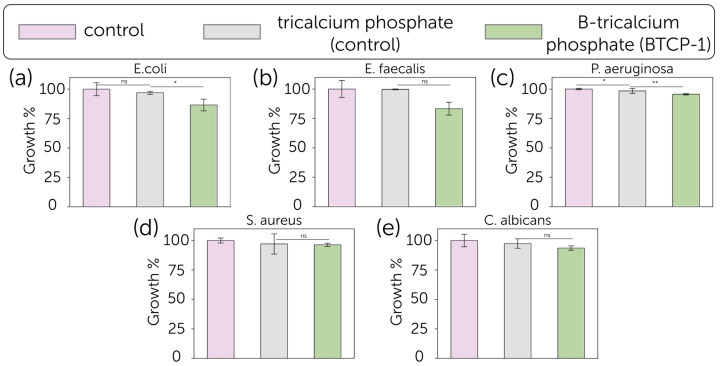
Growth (%, bars) and standard deviation (SD) for (**a**) *E. coli*, (**b**) *E. faecalis*, (**c**) *P. aeruginosa*, (**d**) *S. aureus*, and (**e**) *C. albicans* cultured in the presence of blank control (pink), pure TCP (grey) and B-TCP (green) (*n* = 3). The reported values were obtained from three independent experiments and expressed as mean percentage values ± SD, compared to growth in the presence of TCP and blank control, considered as a control set at 100%. The p values (One-way ANOVA test) are given as the following: ns, not significant; *, *p* < 0.05; **, *p* < 0.01; compared to positive control.

The osteogenic differentiation potential of MSCs was evaluated in the presence of TCP and B-TCP. Calcium deposits in the extracellular matrix were visualized in red using Alizarin Red S staining. The data demonstrate the differentiation potential of MSCs into the osteogenic lineage in the presence of TCP and B-TCP (Figure 5b,c). In all studied conditions, differentiation and calcium deposition in the extracellular matrix were observed. Specifically, in the presence of TCP, the calcium content in the extracellular matrix was 1.24 (*p* < 0.05 *), higher than that of the control, as shown in Figure 5b. Similar results were observed for biphasic ceramics, revealing the positive effect of boron release on osteogenic activity and mineralization [52,53]. Moreover, the effect of boron on osteogenic differentiation potential has been found to be dose-dependent, which may be of particular interest for further studies on B-TCP ceramics. The cytocompatibility of B-TCP powders was revealed, so the research was further related to the assessment of the antimicrobial performance of B-tricalcium phosphate (B-TCP1 sample) (Figure 6).

The antimicrobial activity of TCP and B-TCP substrates was evaluated against the growth of five microorganisms (*E. coli*, *S. aureus*, *P. aeruginosa*, *E. faecalis*, and *C. albicans*) as shown in Figure 6. The positive control for each experiment consisted of each microorganism grown in the absence of substrates for 24 h at their optimal growth temperature. The growth percentage and standard deviation (SD) were obtained from the mean OD600 of three independent experiments. The B-TCP powder exhibited a modest antibacterial effect against *E. coli*, with 13.5 ± 5.0% growth inhibition, and *E. faecalis*, with 16.7 ± 5.5% inhibition. Regarding the other tested bacteria strains and fungus, the inhibition was not significant. The effect of TCP was less pronounced with respect to B-TCP.

The bacterial growth inhibition results obtained in this work are relatively modest compared to the previously reported data for the boron-substituted tricalcium phosphate, calcium pyrophosphate composite ceramics, that demonstrated the inhibition efficiency up to 46.8%, and calcium magnesium silicate cement [25,34]. In contrast, hydroxyapatite-wollastonite biphasic ceramics showed lower antibacterial activity with inhibition rates of 4.5% against *E. coli* and 7.8% against *S. aureus*, what supports the supposition that the antibacterial activity of boron-contained ceramics was majorly guided by the materials’ solubility (biodegradation rate). Thereby, the solubility of ceramics might play a crucial role in both cytocompatibility and antibacterial performance. However, while obtained dense ceramic samples have shown improved cytocompatibility, their antibacterial activity has been reduced, as more soluble components, such as calcium pyrophosphate addition, can boost the inhibition level. Otherwise, HAp-TCP composites might have had a lower antibacterial performance at the early stages of the experiment due to the HAp’s low solubility.

Compared to other element substitutions, borate-substituted tricalcium phosphate ceramics showed similar antibacterial performance to Sr-containing and Cu, Sr-containing bioceramics at 1% substitution (in comparison to non-substituted material). It is worth noting that a further increase in these element contents led to enhanced antibacterial activity and reduced cytocompatibility, which was 20–50% (of viable cells), while B-TCP has not shown a cytotoxic effect [30,31]. Moreover, borate-substituted tricalcium phosphate bioceramics showed modest antibacterial activity against Gram-negative *P. aeruginosa*, while Zn- and Mn-substituted bioceramics had no effect on *P. aeruginosa* growth [54].

## 3. Materials and Methods

### 3.1. Materials

Powders of calcium oxide (CaO, chemical grade, Sigma-Aldrich, St. Louis, MO, USA), ammonia phosphate dibasic ((NH_4_)_2_HPO_4_, analytical grade, Sigma-Aldrich, St. Louis, MO, USA), calcium carbonate (CaCO_3_, analytical grade, Chimmed, Moscow, Russia), and boric acid (H_3_BO_3_, analytical grade, Labtech, Moscow, Russia) were used as precursors for the boron-substituted tricalcium phosphate solid-state synthesis.

### 3.2. Synthesis of the B-Substituted Tricalcium Phosphate and Ceramic Samples Formation

Two different precursor sets were implemented to synthesize the boron-substituted tricalcium phosphate (B-TCP). The first sample, labeled as *BTCP-1*, was produced via the following reaction (1) that occurred during the heat treatment at 1150 °C for 3 h. Prior to the solid-state synthesis, the precursor powders were mechanoactivated by grounding with corundum balls at a 1:20 ratio in a planetary mill at 1800 rpm as follows:

1.9 CaO + 1.9 ((NH_4_)_2_HPO_4_ + 1.1 CaCO_3_ + 0.1 H_3_BO_3_ → Ca_3_(PO_4_)_1.9_(BO_3_)_0.1_ + CO_2_ + H_2_O (1)

The synthesis of the second sample (*BTCP-2*) involved two separate stages. Brushite precursor powder was synthesized through the precipitation from aqueous solutions of calcium hydroxide and ammonia phosphate dibasic with a Ca/P ratio of 1, according to the following reaction (2) and then dried overnight as follows:

CaO + ((NH_4_)_2_HPO_4_ → CaHPO_4_·2H_2_O + 2 NH_4_OH (2)

Subsequently, the boron-substituted tricalcium phosphate was obtained from the freshly synthesized brushite powder and calcium carbonate via the reaction (3) occurred during the heat treatment at 1150 °C for 3 h with a subsequent mechanoactivation by grounding with corundum balls at 1:20 ratio in a planetary mill at 1800 rpm as follows:

1.9 CaHPO_4_·2H_2_O + 1.1 CaCO_3_ + 0.1 H_3_BO_3_ → Ca_3_(PO_4_)_1.9_(BO_3_)_0.1_ + CO_2_ + H_2_O (3)

To produce ceramic samples, B-TCP powders were pressed into d = 10 mm steel molds using a force of 1 metric ton. Subsequently, the resulting samples were sintered in a chamber furnace at the temperature of 1100 °C for 3 h.

### 3.3. Physical and Chemical Characterization

The phase composition of the powders obtained after the synthesis and sintering was determined by the X-ray powder diffraction (XRD) analysis using a Rigaku D/Max-2500 diffractometer (Rigaku Corporation, Tokyo, Japan) with a rotating anode (Cu–Ka radiation, λ = 0.15418 nm), angle interval 2Ѳ: from 2° to 70° (step 2Ѳ − 0.02°). Phase analysis was performed using the ICDD PDF2 database.

The IR spectra of powders obtained after synthesis and sintering were collected using a Spectrum 3 FTIR spectrometer (Perkin Elmer, Waltham, MA, USA) in ATR mode in the wavenumber range of 4000–520 cm^−1^ with a Universal ATR accessory (diamond/KRS-5 crystal).

Scanning electron microscopy (SEM) images of synthesized powders and ceramic pellets were characterized by SEM on the NVision 40 microscope (Carl Zeiss, Jena, Germany) at the accelerating voltage of 5 kV in a secondary electron imaging mode (SE2 detector). A chromium coating (≤10 nm thick) was deposited onto the surface of the ceramic sample using a Quorum Technologies Q150T ES spraying system (London, UK). The solid-state 31P MAS NMR spectra were acquired by means of a BRUKER AVANCE-II 400 WB NMR spectrometer (Billerica, MA, USA) operating at a magnetic field of 9.4 T using a 4 mm H/X/Y MAS DVT probe (spinning rate is 10.5 kHz). Cross-polarization (CP) and Hahn-echo (HE) pulse sequences were combined with high-power proton decoupling (31P). The 31P 90° pulse was equal to 5.5 μs, with a recycle delay of 300 s. The CP MAS spectra were recorded using the polarization transfer technique with a linearly decreasing pulse on 1H nuclei during polarization transfer (contact time, 2 ms; recycle delay, 2 s). The swept frequency two-pulse phase modulation (SW-TPPM) sequence (τ = 8 μs, φ = 15°) was used for proton decoupling. Concentrated phosphoric acid was used as a chemical shift reference (0 ppm).

### 3.4. Isolation of Mesenchymal Stromal Cells

Mesenchymal stromal cells (MSCs) were isolated from the adipose tissue of a 24-month-old male horse post-slaughter. The adipose tissue was minced and digested at 37 °C with 0.1% collagenase IA (Sigma-Aldrich, St. Louis, MO, USA) for 1 h under gentle agitation. Following digestion, the tissue was centrifuged at 800× *g* for 10 min. The isolated cells were resuspended in a DMEM growth medium (Thermo Fisher Scientific, Altrincham, UK) containing 10% fetal bovine serum (FBS; Thermo Fisher Scientific, Waltham, MA, USA) and seeded in culture flasks at 37 °C with 5% CO_2_.

### 3.5. Cytocompatibility Test

The MTT assay was employed to evaluate the toxicity of TCP and B-TCP. The powders were sterilized by autoclaving at 121 °C for 20 min before use. MSCs at the third passage were detached and seeded in 24-well plates in a growth medium at a concentration of 40,000 cells/mL. After 24 h at 37 °C and 5% CO_2_, the medium was replaced with a growth medium containing 1 mg/mL of either TCP or B-TCP. For the positive control, the medium was replaced with fresh growth medium without the powders. Each experiment was performed in triplicate. Following 24 h of incubation at 37 °C and 5% CO_2_, the supernatant was removed and replaced with 1 mL of MTT solution (0.5 mg/mL in DMEM; Sigma-Aldrich, St. Louis, MO, USA). After 3 h at 37 °C, the MTT solution was discarded and replaced with isopropanol (Sigma-Aldrich, St. Louis, MO, USA) for a 30-min incubation at room temperature. The solubilized formazan was measured at an optical density (OD) of 600 nm using a BioPhotometer (Eppendorf, Hamburg, Germany).

### 3.6. Osteogenic Differentiation Assessment

A concentration of 40,000 cells/mL of MSCs at the third passage was seeded into 6-well plates and incubated at 37 °C with 5% CO_2_. After 24 h, the growth medium was replaced with osteogenic differentiation medium (DMEM with 10% FBS, supplemented with 50 µg/mL of ascorbic acid, 10 mM β-glycerophosphate, and 10^−7^ M dexamethasone; all from Sigma-Aldrich, St. Louis, MO, USA) containing 1 mg/mL of either sterile TCP or B-TCP. The positive control consisted of MSCs grown only in an osteogenic differentiation medium, while the negative control contained only a growth medium. The cultures were continuously exposed to the powders throughout the 3-week osteogenic differentiation period. The incubation was carried out for 3 weeks at 37 °C, with the medium being changed every two to three days. Each experimental trial was performed in triplicate. Calcium deposits were visualized using Alizarin Red S staining (Sigma-Aldrich, St. Louis, MO, USA), highlighting the cells in red. Staining involved fixing the MSCs with 4% paraformaldehyde for 30 min, washing three times with distilled water, and staining with 3% Alizarin Red S solution for 30 min. Following four washes with distilled water, red calcium deposits were evident in the extracellular matrix. Images were captured using an inverted optical microscope (Nikon, Melville, NY, USA, Eclipse TE 2000-U). For quantification of calcium deposits in the extracellular matrix, the stained MSCs were incubated with a solution of 5% sodium dodecyl sulfate in 0.5 M HCl (Sigma-Aldrich, St. Louis, MO, USA) for 30 min. 1 mL of each sample was analyzed for optical density at 490 nm using a BioPhotometer, following the method described by Pang et al. [55].

### 3.7. Antimicrobial Tests

The antimicrobial properties of TCP and B-TCP were investigated using four bacterial species (*E. coli*, *S. aureus*, *P. aeruginosa*, and *E. faecalis*) and one fungal species (*C. albicans*). The powders were autoclaved at 121 °C for 20 min prior to use. The microorganisms were cultured in Brain Heart Infusion (BHI) broth (DIFCO, Sparks, USA) in the presence of 1 mg/mL of either TCP or B-TCP for 24 h at their respective growth temperatures under gentle agitation; bacteria were incubated at 37 °C, while *C. albicans* was incubated at 28 °C. For each trial, the positive control involved culturing each microorganism only in BHI. All experiments were conducted in triplicate. Microbial proliferation was quantified by measuring the optical density at 600 nm using a BioPhotometer (Eppendorf, Hamburg, Germany).

### 3.8. Statistical Analysis

The results of the MTT assay, Alizarin Red S quantification, and microbial growth rates were expressed as the mean of three experiments ± standard deviation (SD) and subjected to statistical analysis. Statistical comparison of average values was performed using the multiple-measure Dunnett test (OriginPro 2016 ver. b9.3, OriginLab). The resulting *p*-values were reported in figures: *p* < 0.05 *, *p* < 0.01 **, *p* < 0.001 *** vs. control.

## 4. Conclusions

Following the mechano-activation stage, the boron-substituted tricalcium phosphate powder was obtained by the solid-state reaction at 1150 °C with the initial Ca/(P+B) ratio of 1.5. The preliminary stage of the brushite synthesis was revealed to increase the hydroxyapatite impurity content in the final powder due to the scarce homogenization. At the same time, the implementation of raw calcium oxide and ammonia phosphate dibasic directly resulted only in a minor HAp presence (about 5 wt%). IR analysis revealed the presence of BO_3_^3−^ in the obtained materials. Moreover, the data correlated with the XRD and showed a significant HAp presence in BTCP-2, while BTCP-1, synthesized from raw precursors, had no HAp-specific lines. ^31^P NMR spectra further proved the B-substitution in the tricalcium phosphate. Obtained ceramic samples were shown to be highly porous, which was promoted by the sufficient gas release during sintering.

The study on the biocompatibility of TCP and B-TCP yielded excellent results both for the MTT assays and the osteogenic differentiation. The boron-substituted calcium phosphate ceramic presented a significant interest due to its potential antibacterial performance. The synthesized B-TCP powder showed 13.5 ± 5.0% growth inhibition of *E. coli* and 16.7 ± 5.5% inhibition of *E. faecalis*.

Therefore, this study presented the approach to the solid-state synthesis of boron-substituted tricalcium phosphate, which can be used for the fabrication of biocompatible ceramic bone substitute scaffolds with modest antibacterial properties.

## Data Availability

The experimental data are available at a reasonable and official level by request to the corresponding authors.

## Abbreviations

The following abbreviations are used in this manuscript:

B-TCP	Boron-substituted tricalcium phosphate
β-TCP	β-tricalcium phosphate
XRD	X-ray diffraction
NMR	Nuclear magnetic resonance
CP	Calcium phosphate
HAp	Hydroxyapatite
PJI	Prosthetic joint infection
FTIR	Fourier-transform infrared spectroscopy
SEM	Scanning electron microscopy
SD	Standard deviation
MSC	Mesenchymal stromal cells
